# Antimicrobial De-Escalation in Critically Ill Patients

**DOI:** 10.3390/antibiotics13040375

**Published:** 2024-04-19

**Authors:** Eloisa Sofia Tanzarella, Salvatore Lucio Cutuli, Gianmarco Lombardi, Fabiola Cammarota, Alessandro Caroli, Emanuele Franchini, Elena Sancho Ferrando, Domenico Luca Grieco, Massimo Antonelli, Gennaro De Pascale

**Affiliations:** 1 Dipartimento di Scienze dell’Emergenza, Anestesiologiche e della Rianimazione, Fondazione Policlinico Universitario A. Gemelli IRCCS, 00168 Rome, Italy; eloisasofia.tanzarella@policlinicogemelli.it (E.S.T.); sl.cutuli@gmail.com (S.L.C.); gianmarco.lombardi@policlinicogemelli.it (G.L.); fabiola.camm@gmail.com (F.C.); alessandro_caroli@yahoo.it (A.C.); emanuele.franchini01@gmail.com (E.F.); domenicoluca.grieco@policlinicogemelli.it (D.L.G.); massimo.antonelli@gmail.com (M.A.); 2 Medical Intensive Care Unit, Hospital Clinic Barcelona, 08036 Barcelona, Spain; elenasanchoferrando@gmail.com

**Keywords:** antimicrobial de-escalation, empirical therapy, antimicrobial stewardship, critical illness, diagnostic tools

## Abstract

Antimicrobial de-escalation (ADE) is defined as the discontinuation of one or more antimicrobials in empirical therapy, or the replacement of a broad-spectrum antimicrobial with a narrower-spectrum antimicrobial. The aim of this review is to provide an overview of the available literature on the effectiveness and safety of ADE in critically ill patients, with a focus on special conditions such as anti-fungal therapy and high-risk categories. Although it is widely considered a safe strategy for antimicrobial stewardship (AMS), to date, there has been no assessment of the effect of de-escalation on the development of resistance. Conversely, some authors suggest that prolonged antibiotic treatment may be a side effect of de-escalation, especially in high-risk categories such as neutropenic critically ill patients and intra-abdominal infections (IAIs). Moreover, microbiological documentation is crucial for increasing ADE rates in critically ill patients with infections, and efforts should be focused on exploring new diagnostic tools to accelerate pathogen identification. For these reasons, ADE can be safely used in patients with infections, as confirmed by high-quality and reliable microbiological samplings, although further studies are warranted to clarify its applicability in selected populations.

## 1. Introduction

Appropriate antimicrobial therapy [1] and adequate control of infection source [2] are pivotal interventions for the effective management of sepsis [3]. The 2021 Surviving Sepsis Campaign (SSC) Guidelines [3] recommended a timely and broad-spectrum empirical antimicrobial therapy targeting all suspected potential pathogens implicated in the pathophysiology of sepsis (within 3 h of recognition: weak recommendation, very low quality of evidence) and septic shock (within 1 h of recognition: strong recommendation, low quality of evidence). However, antimicrobial exposure may influence the host microbiota [4] and environmental ecology, thus promoting the selection and emergence of multidrug-resistant (MDR) pathogens due to their broad-spectrum activity, as well as potentially inadequate dose delivery and a prolonged duration of therapy [5]. Specifically, it has been reported that the risk of new resistance emergence increases for each day of additional exposure to antipseudomonal β-lactam antibiotics ranging from 2% for meropenem to 8% for cefepime or piperacillin/tazobactam [6,7]. For these reasons, the 2021 SSC Guidelines [3] suggested daily assessment for antimicrobial de-escalation (ADE) aiming to discourage clinicians towards both the overuse of broad-spectrum drugs and the adoption of a fixed or prolonged [8] therapeutic regimen duration (weak recommendation, very low quality of evidence).

## 2. General Principles, Definitions, and Epidemiology

### 2.1. Background

In recent decades, several investigations have shed light on the safety and effectiveness of ADE as a strategy for antimicrobial stewardship programs (ASPs). In 2016, Tabah et al. [9] systematically reviewed 14 papers (2 randomized controlled trials and 12 cohort studies) on 2461 patients and reported considerable variability in the definition of the ADE, which limited the pooling of the results and drew firm conclusions on this topic. More recently, De Bus et al. conducted the DIANA study [10], which was a multicentre, prospective, observational investigation that involved 1495 patients from 152 ICUs in 28 countries and aimed to describe the incidence of ADE and clinical cure on day 7 (primary outcomes), as well as to clarify the patient, infection, and treatment characteristics associated with ADE. In this study, ADE was defined as follows: (1) discontinuation of one or more antimicrobials of the empirical combination therapy that were no longer necessary for the treatment of the infection within the first 3 days of initiation; (2) replacement of an antimicrobial agent with another drug with a narrower spectrum of activity within the first 3 days of empirical therapy. This study revealed that empirical therapy infrequently underwent ADE in critically ill patients (240 patients, 16%) and consisted mainly of the discontinuation of one or more components of combination therapy (125 patients, 52%), followed by replacement of antimicrobial with another drug (31 patients, 13%), and rarely, both approaches (31 patients, 13%). These results were in contrast with previous reports in which ADE ranged between 25% and 81% [9,11], possibly due to the inclusion of patients with lower illness severity compared to the DIANA study, different definitions, and timing of ADE.

To overcome controversies surrounding the definition of ADE and provide evidence-based guidance for clinicians, the ESICM/ESCMID societies formed a task force of 16 international experts with the aim of formulating a position statement [12] to orient future research on ADE. In this document, published in 2020, ADE was defined as follows:

1. Replacing broad-spectrum antimicrobials with agents characterized by a narrower spectrum or a lower ecological impact;
2. Stopping components of an antimicrobial combination:
  a. Stopping an antimicrobial agent prescribed in combination therapy to provide double coverage for certain pathogens;
  b. Stopping an antimicrobial agent prescribed to cover pathogens that are not isolated from microbiological cultures.

The definition of ADE did not include the early discontinuation of all antimicrobial therapies if the infection was ruled out or a short course of antimicrobial therapy was preferred by clinicians. Although this was the first international position statement on ADE, the quality of evidence supporting this definition is low, and future research is strongly advocated to provide solid results that may guide clinical practice on this strategy.

### 2.2. Determinants

In their seminal systematic review and meta-analysis, Tabah et al. [9] identified several factors directly correlating with ADE, which were as follows: microbiological documentation (e.g., pathogen growth from blood culture or respiratory sampling); initially appropriate antimicrobial therapy; use of more than one agent and companion drugs; lower baseline severity; clinical resolution at the time of culture results; compliance with guidelines to start antipseudomonal beta-lactams in neutropenic patients and recovery from neutropenia in neutropenic patients. Conversely, the documentation of infections caused by MDR pathogens, multiple concomitant infections, and infections with a high risk of undiagnosed pathogens (e.g., intra-abdominal infections) were negatively associated with ADE. However, the results of this systematic review and meta-analyses were potentially biased due to the inclusion of “early discontinuation of antimicrobials” as part of the ADE definition, which is in contrast with the 2020 ESICM/ESCMID position statement [12]. Similarly, the DIANA [10] study found that the ADE group was characterized by higher rates of microbiological confirmation (74.2% vs. 48%, respectively), bacteremia (32.5% vs. 14.1%, respectively), the and need for source control (27.1% vs. 20.6%, respectively) compared to the non-ADE group. Although the ESICM/ESCMID panel [12] made no recommendation on the use of biomarkers to guide ADE, a systematic review and meta-analysis [13] of 10 randomized trials including 3489 adult septic patients reported shorter antimicrobial therapy duration in the PCT-guided group compared to controls (7.35 vs. 8.85 days, respectively; weighted mean difference, −1.49 d; 95% CI, −2.27 to −0.71; *p* < 0.001), in the absence of any effect of Intensive Care Unit (ICU) length of stay (LoS) and mortality.

### 2.3. Safety and Effectiveness

The ADE is likely to be safe, and recent systematic reviews and meta-analyses reported no clinical complications or adverse events associated with this strategy [3,9], both in bacterial and fungal infections [14]. Furthermore, Tabah et al. [9] found that the pooled effect of ADE on mortality is protective (RR 0.68, 95% CI 0.52–0.88, *p* = 0.04), with no impact on the duration of antimicrobial therapy or ICU LoS. These results were partially in line with those of Leone et al. [15], who conducted a multicentre randomized non-inferiority trial of 116 patients with severe sepsis and found that ADE had no impact on ICU LoS. In this study, the comparison between the non-ADE and ADE groups showed less frequent superinfections (11% vs. 27%, respectively, *p* = 0.03) and longer antimicrobial therapy in the latter (9.9 ± 6.6 vs. 14.1 ± 13.4 days, respectively, *p* = 0.04), although this difference was not confirmed by non-parametric test (9 vs. 7.5 days, respectively, *p* = 0.03). Conversely, a single-center, retrospective, observational study [11] that included 478 anti-pseudomonal antibiotic prescriptions reported a non-significant difference in the emergence of resistance to the initial beta-lactam antibiotic on day 14 between the ADE and non-ADE groups (30.6% vs. 23.5%, respectively, *p* = 0.22). Likewise, no difference was observed in terms of the selection of MDR pathogens between ADE and non-ADE groups (23.5 vs. 18.6, respectively, *p* = 0.35), which is in agreement with previous investigations [15,16,17]. Recently, the DIANA study [10] found that the unadjusted 28-day mortality was similar between the ADE group and non-ADE groups (15.8% vs. 19.4%, *p* = 0.27; RR 0.83 [95% CI 0.6–1.14]), with a numerically lower emergence of MDR pathogens among those who received ADE compared to those who did not receive ADE (7.5% vs. 11.9%, respectively, *p* = 0.052). However, the results of this study were quite limited by the heterogeneous patient population and different ASPs [10].

In this setting, it is worth noting that there is no high-quality evidence investigating the effect of ADE on the physiological outcomes and prognosis of critically ill patients, which remains mainly represented by observational studies biased by unmeasurable confounding factors [18]. Moreover, the direct relationship between ADE and improved outcomes (e.g., mortality and emergence of MDR pathogens) may be potentially explained by the selection bias of preferentially performing ADE in patients at low risk of mortality, characterized by improved severity scores with no documented MDR infections, which may alter the reliability of regression analyses with consequent misleading results.

## 3. The Role of Diagnostic Work-Up

### 3.1. The Importance of Appropriate Sampling

The identification of the source and the causative agent of infection is the cornerstone of ADE. Before starting antimicrobial therapy, obtaining samples from all sites considered to be potential sources of infection should be a general rule, if there is no substantial delay in the initiation of antimicrobials. The SSC international guidelines suggest about 45 min as an example of what may be considered an adequate maximum time to obtain cultures [3].

The risk–benefit balance should always be considered, especially in patients with a high risk of death. However, sterilization of cultures can occur within minutes to hours after the first dose of an appropriate antimicrobial therapy, and this issue could reasonably discourage clinicians from ADE or, even worse, lead to an inappropriate discontinuation of antimicrobial therapy.

In addition to increasing the yield of cultures and facilitating ADE, starting antimicrobial therapy immediately after obtaining cultures has been proven to improve outcomes [19].

In addition to this, it is always essential to contextualize the microbiological results to avoid inappropriate changes in antimicrobial therapy.

Cultures may be obtained from the blood, respiratory tract, urine, skin, soft tissue, bone, joint, cerebral spinal fluid, and stool. Some of these sites are colonized with bacteria, which means that the latter are concentrated enough to be detected, but they are not responsible for the signs or symptoms. Other sites (i.e., blood and cerebral spinal fluid) are considered sterile, but the collection of the culture can be contaminated and lead to false results. Unlike colonization, in this case, the microorganism is introduced into the analyzed specimen from another site (i.e., normal skin bacteria into blood cultures) or during laboratory processing.

Distinguishing colonization and contamination from infection is critical for accurately interpreting microbiological results and avoiding unnecessary antimicrobial therapy.

In this context, it is important to consider that all samples are not equally reliable; positive blood cultures, if properly collected, are more relevant than other samples.

For reliable sampling, blood cultures should be collected following rigorous skin disinfections, using two sets of aerobic and anaerobic bottles, each filled with about 10 mL of blood. If a microorganism which is most likely a contaminant is isolated, repeating cultures before making any consideration on ADE is recommended [20].

Conversely, the interpretation of respiratory samples is the most challenging in discriminating between infection and colonization.

The respiratory specimens should be collected within 3 days of symptom onset and no later than 7 days, since the amount of bacteria decreases 72 h after clinical onset. The specimens available from the lower respiratory tract include spontaneous or induced sputum, endotracheal aspirates, bronchoscopic or blind bronchoalveolar lavage (BAL), and a protected specimen brush (PSB) [21].

Given the possibility of contamination by the commensal microbiota of the oropharynx, BAL represents the most reliable technique for pathogen identification.

For adequate sampling, sequential aliquots of normal saline totaling at least 100 mL should be instilled, with at least 30% returned for optimal sampling [22].

The quality of a lower respiratory specimen should also be evaluated by assessing the number of polymorphonuclear and squamous epithelial cells in a Gram-stained smear of the specimen [23]. The samples of sputum and tracheal aspirates are considered of high quality if there are <10 squamous cells and >25 leukocytes per optical microscopy field [24].

Quantitative cultures are needed for the diagnosis of pneumonia. The identification of ≥10^6^ CFU/mL in the tracheal aspirate and ≥10^5^ CFU/mL in the BAL is associated with an active infection, while lower counts represent possible contamination. The detection of 10^4^ to 10^5^ CFU/mL in a BAL sample constitutes a “gray zone” [25].

### 3.2. The Role of Rapid Diagnostic Techniques

According to the current guidelines, ADE should be performed as early as possible, within 24 h of definitive culture results and antibiogram availability [12]. However, since rapid diagnostic techniques (RDTs) are becoming more widespread, waiting for up to 5 days for definitive results of conventional bacteria identification can be considered an excessive length of time.

To date, RDTs have been mostly investigated as a tool for targeting the initial antimicrobial therapy, rather than supporting the clinicians on ADE. However, some retrospective studies on blood cultures and RCTs on respiratory samples have recently been published.

In 2018, Claeys et al. developed an antimicrobial stewardship (AMS)-driven treatment algorithm that included the use of RDTs to detect Gram-negative bacteremia [26]. The authors reported that this approach would have resulted in 14.4% appropriate ADE in a cohort of 188 critically ill patients. However, the rates of *E. Coli* or *Klebsiella* spp. resistant to third-generation cephalosporins (>15%) limited ADE based on RDTs due to resistance mechanisms potentially mediated by non-CTX-M β-lactamases. On top of that, the same authors [27] compared three groups: patients with at least one positive blood culture for whom antimicrobial therapy was not oriented by RDT (pre-RDT/AMS group), patients for whom antimicrobial therapy was oriented by the RDT results without any AMS (the post-RDT/pre-AMS group), and patients treated according to both the RDT results and AMS (the post-RDT/AMS group). Remarkably, ADE occurred more frequently (45.2%) in the pre-RDT/AMS group than in both the post-RDT/pre-AMS (31.7%) and the post-RDT/AMS (39.1%) groups (*p* = 0.018). However, the time to ADE (about 60 h) did not significantly change between the study groups (*p* = 0.47).

In 2021, Giannella et al. conducted a systematic review and meta-analysis of 14 papers to assess the effect of another RDT (T2Dx, T2 Biosystems, Lexington, MA, USA) on patient outcomes compared to standard care, which was represented by conventional blood cultures (BC) [28]. The authors found that the mean time to ADE was faster with RDT vs. BC (*p* = 0.02), although heterogeneity among the studies was high, and the definition of ADE differed from the ESICM/ESCMID position paper [12].

In the context of the implementation of their routine clinical use, RDTs have also been increasingly applied to respiratory samples [29,30].

The impact of RDT on ADE in critically ill patients with pneumonia (CAP/HAP/VAP) was recently investigated by Poole et al. using the BioFire FilmArray Pneumonia panel (Biofire diagnostics LLC, Salt Lake City, UT, USA), which detects 27 bacteria and viruses and seven genetic markers of resistance in around 70 min. This pragmatic randomized controlled trial (RCT) compared two populations: the intervention group, whose lower respiratory tract samples were analyzed immediately with RDT, and the control group, whose microbiological investigation was at the discretion of the responsible clinical team. Interestingly, what they found was that 42 of the 100 patients in the intervention group had result-directed ADE compared with 8 of the 98 patients in the control group (*p* < 0.0001). Furthermore, the time to ADE was significantly shorter in the intervention group compared with the control group (5 h vs. 46.5 h, *p* < 0.0001). Regrettably, this study was not specifically powered to detect differences in the clinical outcomes [31].

In 2022, Darie et al. focused on the application of RDT in patients at risk of Gram-negative bacterial pneumonia. They divided the study population into two groups: all patients had respiratory samples assessed by conventional microbiology, while those in the PCR group had samples assessed by multiplex bacterial PCR for Gram-negative bacteria (Unyvero Hospitalized Pneumonia Cartridge; Curetis, Holzgerlingen, Germany). They observed a decrease of 45% in the duration of inappropriate antibiotic therapy in the PCR group. Furthermore, in the PCR group, the multiplex bacterial PCR results supported ADE in 66% of the patients [32]. These results can be easily explained by the faster turnaround times offered by RDT, which allows clinicians to promptly shift from empirical broad-spectrum therapies to a tailored therapeutic approach.

In light of this, although no recommendations have already been made regarding ADE and RDTs [12], the latter are increasingly emerging as useful, easy-to-read, stewardship tools for ICU patients. However, further studies are advocated to clarify the impact of these strategies on ADE.

## 4. Special Conditions

### 4.1. Neutropenic Patients

Prompt and appropriate antimicrobial therapy is essential to improve outcomes in patients with malignancies and severe immunosuppression [33,34]. In fact, more than 60% of patients undergoing chemotherapy and hematopoietic stem cell transplantation develop febrile neutropenia, defined as body temperature ≥ 38 °C and absolute neutrophil count < 0.5 × 10^9^/L [35]. These patients are often colonized with MDR pathogens and are particularly susceptible to developing infections, so clinicians are often prompted to prescribe extended courses of broad-spectrum antimicrobial therapies, contributing to the spread of antimicrobial resistance [36]. Hence, ADE as a part of antimicrobial stewardship algorithms is increasingly considered a main intervention even in immunocompromised patients [33].

It is against this background that the 2011 European guidelines for empirical antibacterial therapy in febrile neutropenic patients (ECIL-4) recommend ADE when pathogens and their in vitro susceptibility are documented, regardless of the severity of clinical presentation (i.e., sepsis and septic shock). Conversely, when negative or unreliable cultures are collected, no change in the empirical therapy is suggested [34].

Despite the lack of high-quality evidence provided by RCT, the safety and feasibility of ADE have been investigated in several observational studies. In a non-intensive care setting, Verlinden et al. reported no increase in septic shock (3.7% vs. 3.0%; *p* = 0.47) and infection-related ICU admission (3.1% vs. 3.3%; *p* = 0.85) in 958 patients with febrile neutropenia when ECIL-4 recommendations were integrated into a clinical algorithm [37]. Similarly, Alegria et al. showed in a quasi-experimental study of 163 patients that re-evaluation and ADE, according to ECIL-4 guidelines, did not impact mortality (15% vs. 11.3%; *p* = 0.76) [38]. Finally, Rainess et al. found no differences in the incidence of recurrent fever or antibiotic escalation when ADE was applied (35.3% vs. 39.3%; *p* = 0.83), whereas the median length of broad-spectrum antibiotics was significantly lower in the ADE group (3 days vs. 8 days, *p* < 0.001), proving its efficacy and safety in these patients [39].

In an ICU setting, Mokart et al. described ADE strategies in a prospective observational study of 101 neutropenic ICU patients. They reported a cumulative incidence of ADE of 44%, with 68% of the patients still neutropenic. They also observed higher rates of ADE in patients with microbiological documentation and adequate empiric therapy (55% vs. 32%), as well as a longer duration of antibiotics in those who underwent de-escalation (9 days vs. 5 days; *p* = 0.005). ADE was not associated with higher odds of death within the first 30 days (HR 0.51 (95% CI 0.20–1.33)) [40]. More recently, Contejean et al. conducted a study in which a pre-intervention period was compared with a post-intervention period after the implementation of local guidelines based on the ECIL-4 recommendations. No differences in negative outcomes were found (5.1% vs. 3.7%; *p* = 0.59). In addition, they observed a significant decrease in carbapenem and glycopeptide use without an increase in other antibiotic consumption. A limitation of this study is that patients with allogeneic stem-cell transplants were not included [41]. Similarly, La Martire et al. reported a consistent reduction in carbapenems and anti-Gram-positive agents administered with a reduction in antibiotic expenses when ECIL-4 recommendations were adopted [42].

Therefore, as confirmed by Tabah et al.’s position statement [12], ADE seems to be safe in high-risk neutropenic patients and it should be part of daily antimicrobial stewardship.

### 4.2. Intra-Abdominal Infections

Intra-abdominal infections (IAIs) are a frequent cause of sepsis, septic shock, and mortality [43]. Cornerstones of the treatment of IAIs include early identification, effective source control, and prompt antimicrobial therapy [44].

IAIs are often polymicrobial, with a broad spectrum of different pathogens, including Gram-positive and Gram-negative aerobic and anaerobic bacteria and fungi. A recent multicentric observational study found no difference in MDR prevalence between community- and hospital-acquired IAIs [45]. Hence, empirical therapy is often based on the use of combined broad-spectrum antibiotics. From this perspective, antimicrobial stewardship programs and ADE could be relevant.

In 2016, Montravers et al. studied 311 critically ill patients with healthcare-associated IAI in a prospective single-center observational study. De-escalation was not associated with a difference in mortality on day 28 (HR 0.57; 95% CI (0.25–1.28)). Adequate empiric therapy was identified as the main determinant of ADE, while the presence of MDR and non-fermenting Gram-negative bacteria were the main risk factors for no de-escalation [46]. Nevertheless, many authors identified IAI as a deterrent to de-escalating broad-spectrum antimicrobial therapy. For example, a recent DIANA multinational study reported that in a subgroup of 272 IAI cases, ADE was performed in only 14% of cases [10]. In addition to this, a retrospective analysis by De Waele et al. identified IAI as a leading factor for not performing ADE in patients undergoing empirical meropenem treatment. However, the authors only considered patients treated with meropenem, without considering other forms of ADE, such as the withdrawal of an empiric glycopeptide or the replacement of an antipseudomonal penicillin with a narrower-spectrum penicillin [47].

This uncertainty about de-escalation in treating IAI could be due to several factors. First, it can be the consequence of doubts regarding inadequacy or timing of source control, which is the most relevant intervention in these patients [48]; second, IAIs are often polymicrobial, and this represents a limitation concerning the reliability of cultures and the correct identification of the causative pathogen, especially in immunocompromised patients [48]. Finally, targeted therapy must reach an adequate target site concentration; however, in settings of peritonitis and abdominal sepsis, pharmacokinetics might be altered, leading to suboptimal antibiotic concentration at the infection site [49]. Regarding this, the increasingly widespread use of therapeutic drug monitoring (TDM) of antimicrobial agents could help to safely titrate the drug dosage to reach optimal exposure.

Although the quality of evidence supporting this intervention is low, ADE in the context of severe IAIs might be a feasible practice, according to current position statements [12,44]. It can be safely enforced whether correct, evidence-based, multidisciplinary management (adequate empirical therapy, good quality and reliability of microbiological samplings, and effectiveness of source control) has been properly implemented from the start.

### 4.3. Antifungal De-Escalation

According to the current position statement, an ADE strategy for systemic antifungal therapy appears to be safe, and it is strongly recommended after the clinical and microbiological resolution of invasive candidiasis [12]. It is defined as the discontinuation of initial antifungal treatment within the first 5 days from the beginning, or the replacement of the initial antifungal (except fluconazole) with a narrower-spectrum antifungal [50].

Antifungal ADE is particularly challenging due to the relatively reduced choice of classes of antifungal drugs and limited clinical expertise compared to antibiotics.

Echinocandins are recommended as the first-line therapy in critically ill patients with sepsis and suspected or documented invasive candidiasis. In fact, they are fungicidal and more reliable than fluconazole as empirical therapy [51].

The same guidelines also recommend de-escalating from echinocandin to fluconazole when patients with negative repeat blood cultures are clinically stable and have isolates susceptible to fluconazole [51]. Despite this, even in contexts with more expertise in antifungal therapy, the rate of de-escalation has been shown to be around 20%, with some studies reporting up to 61% of patients on echinocandins that were not switched to fluconazole despite their *Candida* isolates being fully susceptible [52,53].

In this context, the proportion of *Candida* isolates non-susceptible to echinocandins is increasing, and several outbreaks of multi-drug resistant *Candida auris* infections have been reported worldwide, making the decrease in the exposure to these antifungals strongly desirable [54].

Currently, available fungal-specific biomarkers and new RDTs may help clinicians to achieve this goal. Among the non-culture-based diagnostic tests, (1,3)-β-D-glucan (BDG) is commonly used as a biomarker for the prompt diagnosis of invasive candidiasis. Although false-positive results have been documented in many situations, decreasing the overall specificity (60%) and positive predictive value (<15%) of the test in patients at low-intermediate risk, BGD has been demonstrated to be superior to colonization indexes, with high sensitivity (74–86%) and negative predictive value (>95%), in settings with low pre-test probability (IC rate < 5%) [55,56].

Finally, *Candida* T2 Magnetic Resonance (T2MR) is a promising resource, which could shorten the time to Candida detection in bloodstream infections. Its main limitation is that, to date, only five *Candida* spp. have been included in the panel, and its accuracy and role as an AMS tool should be further evaluated [57].

## 5. De-Escalation as Antibiotic Stewardship Tool

In recent decades, antibiotic resistance has been defined as a global emergency, and antibiotic stewardship programs have been pointed out as crucial tools to combat it [58].

Nevertheless, there is still no consensus on the definition of antibiotic stewardship, nor on the elements that must be involved, and it is generally defined as a comprehensive set of actions that promote the responsible use of antimicrobials, with the final aim of reducing ecological pressure [58]. De-escalation is widely recognized as a key aspect of this process, as it decreases microorganism exposure to antibiotics, reduces side effects, and avoids the development of new mechanisms of antibiotic resistance.

Current guidelines declare that the ADE strategy is safe for patients’ outcomes with a moderate quality of evidence and, regarding the issue of culture-negative infections, recommend investigating an alternative non-infectious diagnosis by discontinuing all or part of the antibiotic regimen. Beyond this, there has been no assessment of the effect of de-escalation on the development of resistance [12].

The first observational study designed to investigate the effect of antibiotic de-escalation on microorganism resistance reported a longer antibiotic course associated with de-escalation (8 days) compared with continuation of therapy (5 days) and a higher total antibiotic consumption during ICU admission, concluding that prolonged antibiotic treatment may be a side effect of de-escalation [11].

In contrast, antimicrobial stewardship programs have also been associated with a significant decrease in the prescription of specific antibiotic classes (penicillin plus β-lactamase inhibitors, linezolid, cephalosporins, and aminoglycosides), with no deleterious effects on patient mortality or length of stay [59].

In 2017, an observational before–after study on carbapenem restriction reported a significant reduction in the overall carbapenem consumption from 28.44 to 11.67 DDDs/1000 patient days [60]. In addition, De Bus et al. reported a high number of prolonged treatments where de-escalation was not applied (67%) despite being microbiologically possible [11], which aligns with the growing awareness that ADE is generally not given sufficient consideration in daily clinical practice [61].

Among the different clinical processes and decisions, identifying the pathogen responsible for infection, especially in critically ill patients, is the cornerstone of ADE. Indeed, the success of antibiotic stewardship relies on the accurate interpretation of microbiological results in the context of the clinical presentation of infection, which is a highly complex challenge in routine practice [18].

In this context, multifaceted interventions are the cornerstone for achieving the fundamental goals of antibiotic stewardship programs, including improving outcomes and decreasing therapy-related toxicities [61]. Artificial intelligence may also be of support using dashboards that may capture data from medical records and microbiology systems, thus providing all the needed information in a real-time and easily accessible way. This integration of general patient characteristics, dynamics of local resistance patterns, antibiotic consumption, and adherence to protocols may represent a ‘game changer’ in the decision-making process of healthcare professionals [18,61].

Given all the above considerations, it is a matter of fact that intensivists, as part of a multidisciplinary team, should be at the forefront for the development, optimization, and promotion of treatment plans for severe infections and sepsis, playing a crucial role in the AMS initiative.

## 6. Conclusions

As a cornerstone of AMS strategies, ADE is a widely used practice in precision medicine to decrease the emergence of resistance to antibiotics and antifungals. Our results confirm that it is safe and can be considered an efficient tool in the context of an emerging need for reducing the overall antimicrobial exposure and selection pressure, especially in critically ill patients.

In this context, appropriately collecting and interpreting cultures, avoiding delays in effective antimicrobial therapy, and properly implementing biomarkers and new RDTs can help to achieve concrete results in terms of AMS.

However, although there is a strong physiological and clinical rationale for the safety and effectiveness of ADE [12,18], high-quality evidence investigating its effects on prognosis and physiological outcomes is lacking.

Therefore, further studies are advocated to clarify the most optimal strategies for ADE and its clinical and ecological role in the daily practice of ICU patients with severe infections.

## Data Availability

Not applicable.
